# pH-Responsive DNA Motif: From Rational Design to Analytical Applications

**DOI:** 10.3389/fchem.2021.732770

**Published:** 2021-08-11

**Authors:** Lin Lin Zheng, Jin Ze Li, Ying Xu Li, Jian Bang Gao, Jiang Xue Dong, Zhong Feng Gao

**Affiliations:** ^1^ Shandong Province Key Laboratory of Detection Technology for Tumor Makers, Collaborative Innovation Center of Tumor Marker Detection Technology, School of Chemistry and Chemical Engineering, Feixian Campus, Linyi University, Linyi, China; ^2^ College of Chemistry and Environmental Science, Key Laboratory of Analytical Science and Technology, Hebei University, Baoding, China

**Keywords:** pH-responsive, DNA molecular devices, triplex DNA, i-motif, A^+^-C mismatch

## Abstract

pH-responsive DNA motifs have attracted substantial attention attributed to their high designability and versatility of DNA chemistry. Such DNA motifs typically exploit DNA secondary structures that exhibit pH response properties because of the presence of specific protonation sites. In this review, we briefly summarized second structure-based pH-responsive DNA motifs, including triplex DNA, i-motif, and A^+^-C mismatch base pair-based DNA devices. Finally, the challenges and prospects of pH-responsive DNA motifs are also discussed.

## Introduction

Deoxyribonucleic acid (DNA), as the main carrier of genetic information for living organisms, has been widely studied (Zhang and Seelig, 2011; Lu et al., 2013). Because of its thermodynamic programmability, high structural features, facile synthesis, and possible conjugation to a host of molecules and nanodevices, DNA has presented an impressive potential to engineer a variety of molecular devices with applications ranging from molecular sensors to therapeutic tools (Ablasser, 2021; Kremarová et al., 2021). Thanks to those crucial properties, the functionalized DNA devices have been studied. The precise definition of the operational characteristics, especially the sensitivity, is indispensable for the development of DNA-based molecular machines to regulate the vital biological processes and sense (Ranallo et al., 2015; Manish et al., 2019). The degree of their response sensitivity to different environmental stimuli, such as metal ions (Collie and Parkinson., 2011), pH value (Gehring et al., 1993), light (Beharry et al., 2011), and enzymatic activities (Wickham et al., 2012), is determined by the magnitude of their folding structure (Simon et al., 2014).

**FIGURE 1:**
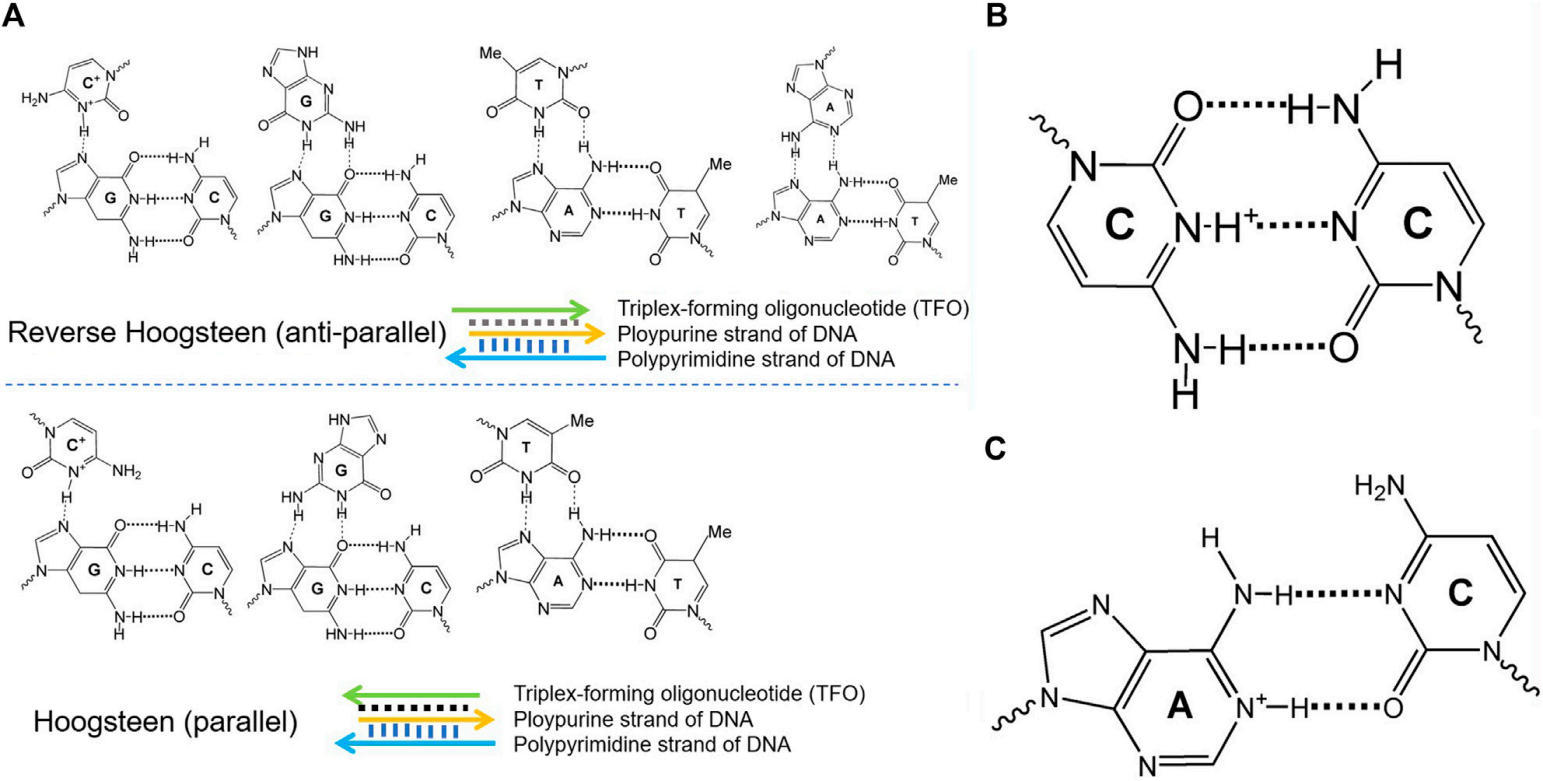
Different conformational DNA structure configurations. **(A)** Different configurations of the parallel and antiparallel triplex DNA. **(B)** The key element C^+^-C in the i-motif DNA. **(C)** The protonation center of A^+^-C in the pH-dependent DNA motif.

DNA molecular devices are DNA self-assemblies induced by external stimuli, perform quasi-mechanical movement at the micro-nano scale, and have attracted increasing attention in the fields of biosensing, drug delivery, and biomedical detection (Kay and Leigh., 2015; Abendroth et al., 2015; Tani et al., 2021). By taking advantage of the high designability and the versatility of DNA chemistry, several groups have recently developed pH-dependent DNA-based nanodevices (Fu et al., 2019). Such functionalized DNA devices typically exploit DNA secondary structures that display pH dependence because of the presence of specific protonation sites. These structures include triplex DNA, i-motif, and A^+^-C mismatch base pair-based DNA, displayed in Figure 1. The pH-responsive property is highly due to the protonation of adenine and cytosine in the A^+^-C mismatch pair, triplex DNA, and i-motif, respectively (Soto et al., 2002). Different physical methods such as nuclear magnetic resonance (NMR), calorimetry, X-ray fiber, UV/Vis spectroscopy, and diffraction methods have been applied to characterize the thermodynamic stabilities (Plum et al., 1990) kinetic properties (Maher et al., 1990), and structural features (Wärmländer et al., 2003) of the different DNA structures and their relative motifs.

In this review, we mainly described the pH-dependent DNA structures including triplex DNA, i-motif, and A^+^-C mismatched DNA structures and their applications in biosensor, living cells imaging, and accurate regulation of the pH response range. Through the studies of the different pH-dependent DNA structures, we expect that the DNA nanotechnology and its related field will continue to develop rapidly. At a fundamental level, further studies should aim at greater understanding of the conformational transformation mechanisms at the nanometer scale. In terms of applications, we expect that many of these elegant DNA molecular devices will soon be used *in vivo*. These further studies could confirm the power of DNA nanotechnology in biology, material science, chemistry, and physics.

## The Different Conformational pH-dependent DNA Devices

### Triplex DNA Nanodevices

Triplex nucleic acids have recently attracted attention as part of the rich “toolbox” of the structures used to develop DNA-based nanodevices and materials. In the 1980s (Singleton and Dervan, 1992), DNA was first utilized to assemble nanostructures on the basis of the specific base-paring of single-stranded DNA overhangs, and a set of sophisticated DNA-based nanostructures have been rationally designed. Apart from the base-paired duplex structure of oligonucleotides, supramolecular DNA assemblies formed by the interaction of the double-helix DNA strand and an auxiliary single-stranded overhang were used to form triplex assemblies through Hoogsteen interactions, termed triplex-forming oligonucleotide. Different triplex structures between double-helix DNA domains and single-stranded oligonucleotides domains are demonstrated in Figure 1, including the parallel triplex structures composed of C^+^·G-C, G·G-C, and T·A-T and antiparallel triplex structures formed by C^+^·G-C, G·G-C, T·A-T, and A·A-T strands. There are different parameters such as mutations in the triplex domains, the number and kind of the triplex bridges, the pH value, and the presence of binders or ions, which would have an effect on the stability of the triplex DNA nanodevices. With the purpose of building sophisticated and functionalized molecular machines, a host of groups have devoted constant efforts to developing new mechanisms of manipulated structural reconfiguration by controlling those parameters.

**FIGURE 2:**
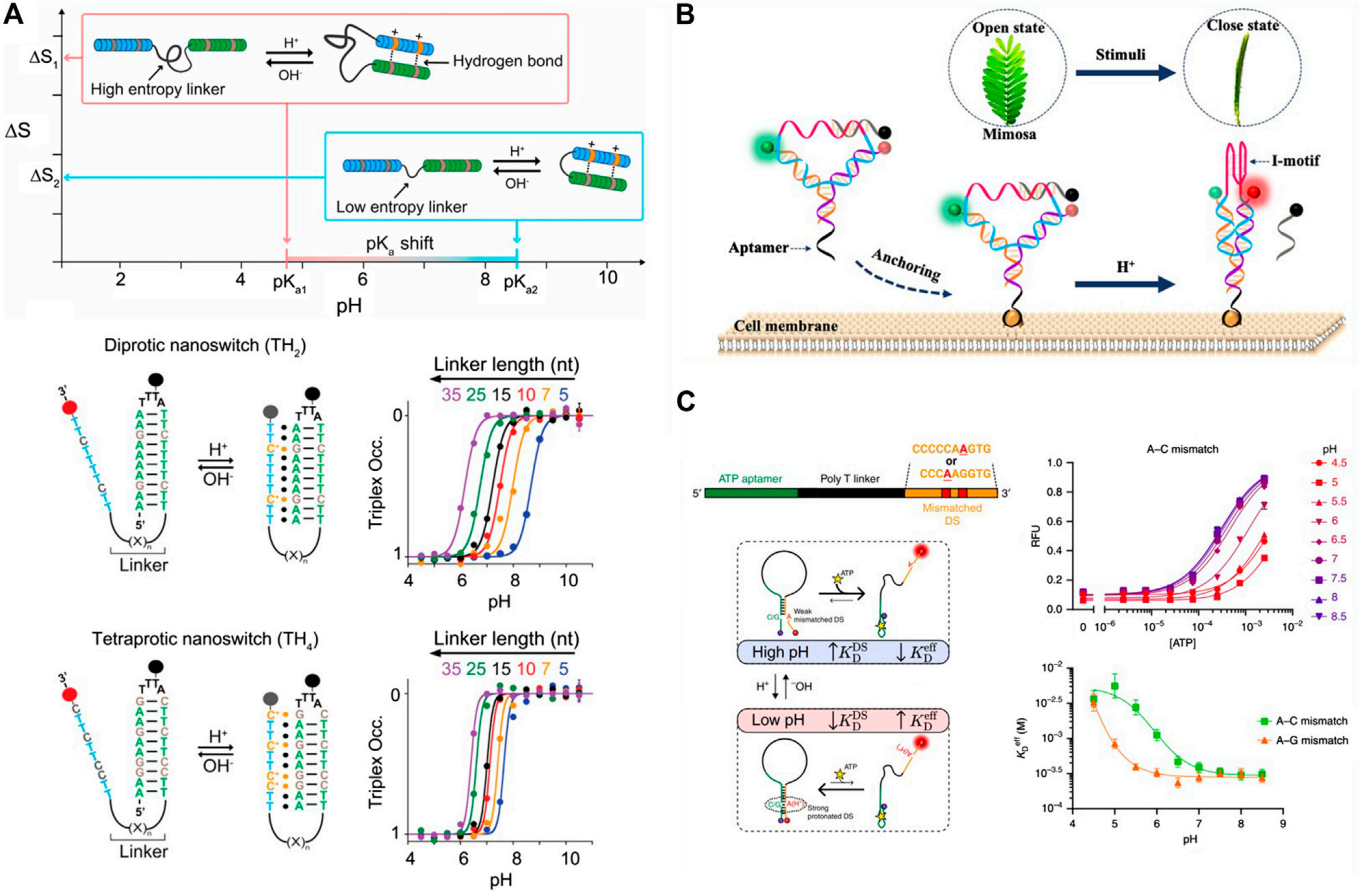
The representative study based on the pH-dependent DNA motif. **(A)** pH triplex nanoswitches with rationally tailored pK_a_ (Mariottini et al., 2019), reproduced with permission. Copyright 2019, Journal of the American Chemical Society. **(B)** Y-shaped DNA nanostructure based on the i-motif DNA (Chen B et al., 2020), reproduced with permission. Copyright 2020, ACS. **(C)** pH-dependent DNA switch containing A^+^-C mismatch pairs (Thompson et al., 2020), reproduced with permission. Copyright 2020, Springer Nature.

Motivated by the above description, some pH-responsive triplex switch has been designed for the rational regulation of the thermodynamic property. Such triplex switch is generally combined with three key elements: pH-dependent protonation center C^+^·G-C, T·A-T, and the linker connecting the single-strand triplex-forming overhang to the hairpin duplex domain. Taking the key elements into account, Mariottini and coworkers demonstrated that the acidic constant (pK_a_) of the pH-dependent nanoswitch could be rationally regulated by the design of the intrinsically disordered domain which connected with the hydrogen-bond-forming regions (Mariottini et al., 2019).To investigate this assumption, a pH-dependent triplex nucleic acid nanoswitch was designed, which can generate an intramolecular triplex conformation through the interaction of Hoogsteen between a single-strand triplex-forming overhang and the hairpin duplex domain, shown in Figure 2A. The gradual increase of the triplex nanoswitch destabilizing pH value could be observed when the linker length is shortened. In a nutshell, they demonstrated that the regulation of the observed pK_a_ is greatly attributed to a purely entropic function of the linker length, which can be weakened by improving the number of protonation centers contained in the single-stranded triplex-forming overhang. All the strategies present a predictable and versatile approach to reasonably regulate the thermodynamics and the kinetics of the synthetic structures and expand the range of their application into the designing of nanomolecular devices with a reversible pH dependence and use for controllable drug delivery.

The triplex DNA structures play a pivotal role in the designing of stimuli-responsive nucleic acid devices. The generation of the metal-organic frameworks and the substrate-loaded microcapsules based on DNA which are unlocked by the pH-stimulated conformation of the triplex nucleic acid structures have been amplified by the triplex DNA structures (Liao et al., 2016; Chen B et al., 2020). In addition, the thermodynamic property of the triplex DNA structures with protonation centers and linker led to the regulation of pK_a_. Despite the reported accomplishment of applying the triplex DNA structures in nanobiotechnology, significant future challenges should be envisaged. Those preliminary results highlight the importance of the DNA triplexes in the future nanomedicine applications.

### I-Motif DNA Structure

In 1993, Gueron and coworkers first reported the i-motif structure (Gehring et al., 1993). They reported that the protonated cytosine-rich (C-rich) sequences could assemble into a four-stranded structure, intercalated motif in an acidic condition, while changing to single strands under basic or neutral conditions. Taking advantage of this finding, the *in vivo* existence of the i-motif structures has been a hot spot. Therefore, the intermolecular assembly among different C-rich strands cannot be easily controlled because of their self-association. Alternatively, different functionalized i-motif DNA units can be put into an interlocked structure to prevent the unessential interstructural association of i-motifs and make them be a potential molecular material for biosensing.

The practicability of approaches for molecular biosensing containing an adjustable dynamic range is important for understanding and controlling the essential biological processes. Nesterova and coworkers reported design strategies of sensitively pH-responsive sensor based on the nucleic acid i-motif (Nesterova and Nesterov, 2014). Through the reasonable manipulation of the i-motif and collaboration of allosteric control elements, they successfully managed to narrow the total response range to 0.2 pH and delivered a transition midpoint with 0.1 pH units precision. By incorporating three vital characteristics of reversibility of the transition range, intrinsic incorporation of i-motif folding, and incorporation adjustment, their presented strategy can be used in pH-sensitive DNA devices and can be applied to expand the manipulation of other four-strand based structures and will promote the development of the ultraresponsive elements for the signaling systems and the artificial molecular devices.

Relying on the pH-dependent i-motif, Chen and coworkers designed a Y-shaped DNA nanosensor, which can be anchored on the surface of the tumor cell by the specific recognition of an aptamer (AS1411), demonstrated in Figure 2B (Chen Y et al., 2020). By labeling pH-independent fluorescence pair on the Y-shaped DNA, the obvious FRET performance could be observed when putting the anchored tumor cell into different pH conditions. In an acidic solution, the C-rich single strand can fold to i-motif structure, and the FRET acceptor is excited. In a neutral condition, the i-motif was opened and the quenching strand significantly decreased the background FRET signal. The Y-shaped DNA sensor displayed a distinct narrow pH response range of 0.5 units, which can sense the tiny variation of the extracellular pH value of the tumor cells. With those advances, the Y-shaped i-motif containing sensor was successfully applied to sensing the MCF-7 cells and displayed a visual fluorescence date. The designed pH-dependent i-motif sensor has a potential for the early diagnosis of cancer and to be used in the demonstration of the cellular physiological progress related to the extracellular pH.

Over the past few years, Yamuna’s group has used the i-motif structure DNA nanodevices as a pH sensor to map the temporal and spatial pH changes inside the living cells and the whole living organisms; with appropriate modifications, this method can also be applied to explore cellular endocytic pathways (Surana et al., 2011; Modi et al., 2013). These studies are crucial for the future research of the DNA nanotechnology. We can design many more molecular devices based on the i-motif structures, which can be used in early cancer diagnostics, molecular operations in the living cells, and drug delivery.

### A^+^-C Mismatched Base Pair-Based pH-dependent DNA Nanostructure

pH dependence DNA nanodevices lay the foundation for various indispensable applications, exclusively based on the structure of the DNA triplex and i-motif. Both structures are dissociated under near neutral solution and are stable in acidic solution with the presence of the interesting protonation site. However, strict sequence requirements are essential for them to own the pH dependence property, which greatly limits the application of the pH-dependent DNA. The i-motif based pH-dependent DNA nanostructure needs to be C-rich, and the triplex pH-dependent DNA nanostructure needs to be neither polypyrimidine nor polypurine. The capability to design general DNA sequences would help us to know more about the physicochemical nature of DNA and create more applications.

Aiming at addressing the limitation mentioned above, as early as 1992, A^+^-C pair was based on the competitive binding of the non-perfectly complementary duplex DNA domain and the complementary strand, and adenine and cytosine were non-perfectly complementary base pairs in the neutral solution (Boulard et al., 1992). But in an acidic condition (e.g., pH = 5.0), adenine is protonated and then is hybridized with cytosine, forming the A^+^-C mismatch base pair. In the near neutral condition (e.g., pH = 8.0), A^+^ is deprotonated and the A^+^-C pair dissociates. Then the A^+^-C mismatch pair-based device is used in rationally designing the programmable pH-responsive DNA devices, shown in Figure 2C (Siegfried et al., 2010; Thompson et al., 2020).

Fu and coworkers reported a universal principle not for triplex but for i-motif forming pH-responsive DNA sequences design (Fu et al., 2019), in which the binding equilibrium changes could be controlled by the pH value of the external solution environment. To demonstrate this mechanism, they first designed a DNA strand M with the length of 20 nucleotides (nt), and a corresponding hairpin strand contained mismatch base pairs to destabilize the hairpin structure, forming MH5. When the pH value of the solution is 8, the hairpin structure of MH5 is opened and hybridized with M to generate a perfectly matched, continuous, 17-bp-long DNA duplex. When the pH value of the solution is 5, the dangling single-stranded overhang of MH5 can be hybridized back to form a hairpin structure and displace strand M. At the end of their work, they applied the design principle to the hybridization chain reaction (HCR), a common method for signal amplification. Their strategy opens new ways to a wide range of potential applications in the DNA-based nanomachines and gives new ideas into nucleic acid structures *in vivo*.

Taking advantage of the high generality and the programmability of the A^+^-C pair-based DNA structure, combining it with other DNA secondary structures, a set of pH-dependent DNA nanodevices would be generated. Taking such structures into account when designing interaction or folding of nucleic acids in such abnormal pH environment, it would greatly facilitate the development of the DNA nanodevices in the biomedical detection and the field of DNA nanotechnology.

## Conclusion

In this review, we summarized the recent research on pH-responsive DNA motifs, including triplex DNA, i-motif, and A^+^-C mismatch base pair-based DNA structures. By rationally modulating the main elements and utilizing the physicochemical nature of the pH sensitive structures, the application of those devices can be commonly used in the biochemical detection and dynamically regulating the pH response range. The variation of the DNA devices’ conditions may lead to revisable conformational changes between the opening and closing states of the pH-dependent nucleic acid structure. In addition to the direct tuning, the pH value can also be fueled by the biocatalytic reaction; for example, urease or glucose oxidase (GOx) has been used in the presence of urea or glucose to motivate the production of the basic or acidic aqueous solution, respectively (Grosso et al., 2015; Gao et al., 2018). Despite the reported accomplishments in applying the pH-responsive DNA motif from rational design to analytical application, some essential future challenges can also be envisaged. For example, the self-assembly of the pH-sensitive DNA-based one-dimensional to three-dimensional nanostructures could generate stimuli-responsive, reconfigurable nanodevices acting as switchable catalytic reservoirs or novel drug carriers. The modification of those DNA nanostructures into single cell and the use of triplex DNA, i-motif structure, and A^+^-C mismatched pairs as functional units modulating intercellular functions would be more and more important.
